# Risk Behaviors and Internet Gaming Addiction Among Nursing Students

**DOI:** 10.1192/j.eurpsy.2026.10881

**Published:** 2026-07-31

**Authors:** R. Nakhli, B. K. Jihene, B. Narjess, G. Naoures, C. Farah, C. Sridi, F. Imen, M. Maoua

**Affiliations:** 1 Occupational Medicine Department, Sahloul University Hospital; 2 Faculty of Medicine of Sousse, University of Sousse; 3 Laboratoire de Recherche (LR19SP03); 4 Clinical Psychology Unit, Occupational Medicine Department, Sahloul University Hospital, Sousse, Tunisia

## Abstract

**Introduction:**

Internet gaming addiction is an emerging mental health issue among young adults, characterized by compulsive use, loss of control, and significant psychosocial impairments. Nursing students, often exposed to academic and emotional stress, may be particularly at risk.

**Objectives:**

To explore health risk behaviors and the prevalence of internet gaming addiction among nursing students.

**Methods:**

A descriptive, analytical, cross-sectional study was conducted from November to December 2024 at the Private Higher Institute of Nursing Sciences (ISIS), Sousse. Data were collected through a self-administered questionnaire and the Lemmens Game Addiction Scale.

**Results:**

Most students (83.7%) perceived internet use as harmful to physical and mental health. Two-thirds (65%) reported difficulty focusing on studies when their smartphone was turned off. Nearly half (45.7%) spent between 2 and 5 hours daily on online games. Popular genres included social/party games, MMORPGs, and competitive games (63%), followed by “Ledo King” (19%). Gaming-related problems were identified in 38.7% of students using the polythetic format of the Game Addiction Scale.

**Conclusions:**

Internet gaming addiction is prevalent among nursing students, with significant academic and psychological implications. Broader studies are needed to better understand behavioral addictions among young adults and inform preventive educational strategies.

**Disclosure of Interest:**

None Declared

